# An Indigenous perspective on ecosystem accounting: Challenges and opportunities revealed by an Australian case study

**DOI:** 10.1007/s13280-022-01746-8

**Published:** 2022-05-27

**Authors:** Anna Normyle, Bruce Doran, Michael Vardon, Dean Mathews, Julie Melbourne, Glenn Althor

**Affiliations:** 1 Fenner School of Environment and Society, The Australian National University, Building 141 Linnaeus Way, Canberra, ACT 2601 Australia; 2 Nyamba Buru Yawuru, 55 Reid Rd, Broome, WA 6726 Australia

**Keywords:** Cultural and environmental management, Ecosystem accounting, Indigenous studies, Natural capital, SEEA

## Abstract

The System of Environmental-Economic Accounting Ecosystem Accounting (SEEA-EA) is widely promoted in environmental and economic policy and management. Unfortunately, the SEEA-EA has not substantively addressed the aspects of accounting that may be of interest to, or used by, Indigenous peoples. We investigate an Indigenous perspective on the potential of the SEEA-EA to support cultural and environmental management through collaborative workshops with managers of Nyamba Buru Yawuru, the Prescribed Body Corporate representing the Yawuru Traditional Owners in Western Australia. Our discussions highlight that while the SEEA-EA may be a valuable tool for empowering Indigenous people and supporting the management of their lands and seas, there are areas where the SEEA-EA needs to be broadened to better reflect cultural values, and the services to ecosystems provided by Indigenous peoples. Embedding Indigenous perspectives into the SEEA-EA would mean that it is of greater use to Indigenous peoples and their representative organisations and ensure that these values are better recognised in the policymaking of government.

## Introduction

The United Nations’ Sustainable Development Goals (SDGs) emphasise the need to include Indigenous practices in the management of cultural, environmental, and economic resources (UNDESA 2016). This is important as more than 40% of Earth’s land area is recognised under some form of Indigenous management or tenure (Garnett et al. 2018). While integrated information is essential for effective management, culturally appropriate quantification and reporting on the values and services provided by Indigenous lands is challenging (Daniel et al. 2012; Chan et al. 2016). This exacerbates the problem of Indigenous values and land management practices being underrepresented within decision-making frameworks (Sangha et al. 2015). Practical engagement with Indigenous peoples is needed to assess how reporting approaches may be adapted to better encompass Indigenous knowledge and perspectives (Bark et al. 2015; Jarvis et al. 2018).

The System of Environmental-Economic Accounting (SEEA) is a framework for the integration of environment and economic information, including cultural ecosystem services. The SEEA formalises the evaluation of the physical and monetary transactions between the environment and economy in a manner that complements the System of National Accounts (Vardon et al. 2016). The SEEA Central Framework (UN 2014a) provides a systematic approach to support information synthesis across multiple sectors, jurisdictions, and sources; with principles and indicators to link environmental assets and services to the economy in both monetary and non-monetary terms (Vardon et al. 2018).

The SEEA Central Framework is limited by its exclusion of most ecosystem assets and services (Obst et al. 2015). To address this, a corresponding ecosystem accounting approach, the SEEA Ecosystem Accounting (SEEA-EA) was developed (UN 2014b) and standardised (UN 2021). By evaluating different effects related to changes in the extent and condition of ecosystem assets and their corresponding service flows over time and space in both physical and monetary terms, ecosystem accounts can be useful for evaluating the aggregate effects of different combinations of management for a particular area (Obst 2018).

Implementation of the SEEA-EA is underway in 24 countries (Hein et al. 2020) with more countries preparing for implementation (UNCEEA 2021). However, no published studies have constructed accounts relevant to the cultural values and services that underpin Indigenous land management and no accounts have been produced by or for Indigenous peoples (Normyle et al. 2022a). While ecosystem accounting may be valuable in providing spatially explicit, regular and accessible information that can support Indigenous owners and managers’ decision-making needs (Stoeckl et al. 2021), this is yet to be demonstrated.

**Fig. 1:**
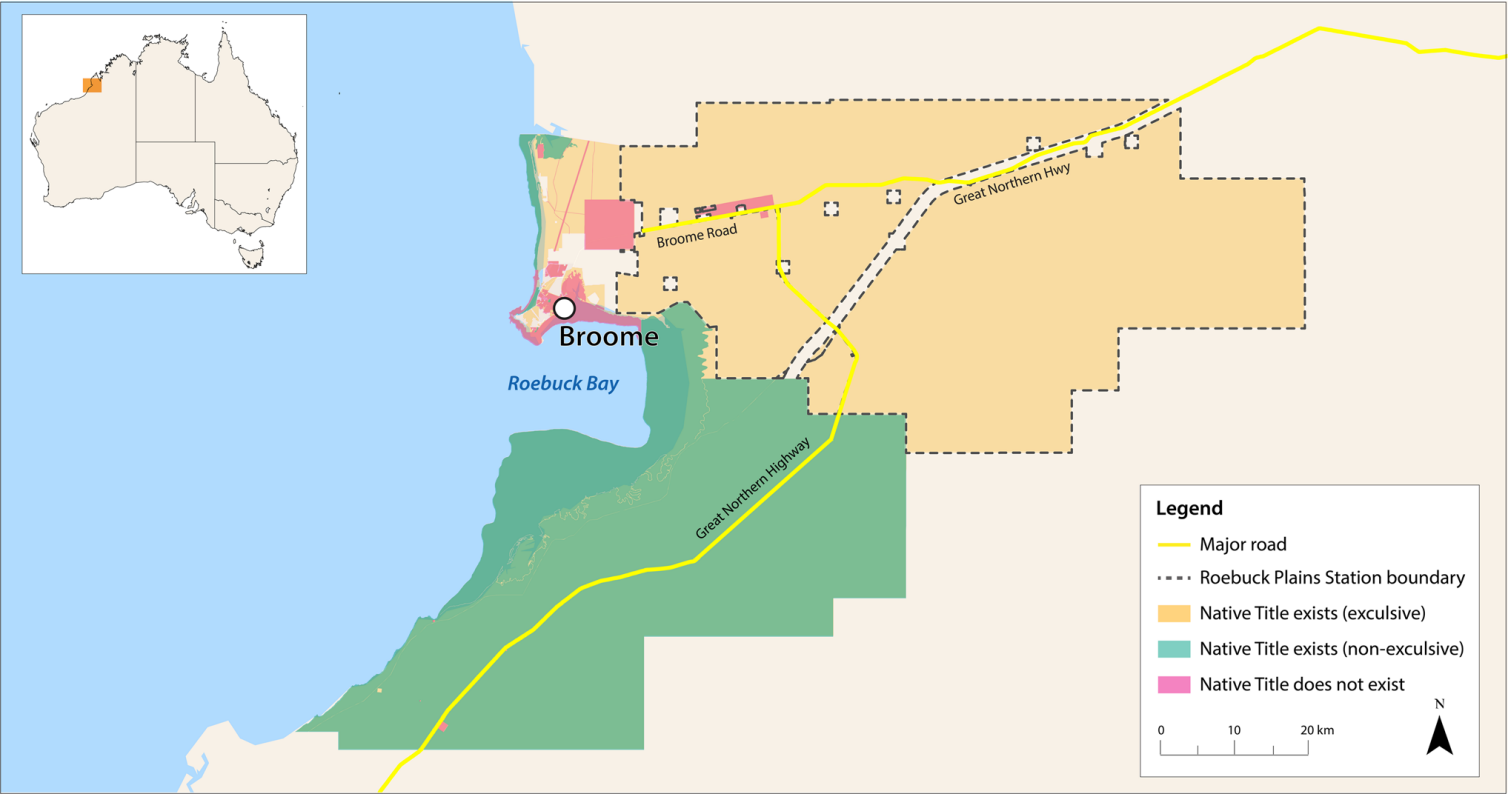
Map of Yawuru Country. The Yawuru people are the traditional custodians of the land and sea Country around Broome, Western Australia

Using a case study from Yawuru Country,^1^ in north-western Australia (Fig. 1), this paper identifies both practical opportunities and conceptual challenges to use of the SEEA-EA to support Indigenous land and sea management. Informed by Yawuru managers’ perspectives on the nature and importance of services to ecosystems and cultural health indicators of Country, we present preliminary approaches for including cultural values within ecosystem accounting and call for further work to engage Indigenous peoples as part of the SEEA research agenda.

### Case study background

In Australia, more than 40% (3 million square kilometres) of the nation’s land and sea territory is recognised under Indigenous title (NNTT 2022). Indigenous cultural and natural resource management (ICNRM) practices including cultural site protection, seasonal burning, weed management, hunting and revegetation have been documented and shown to provide a range of social, environmental and economic benefits (Altman et al. 2007; Jackson and Palmer 2015; Pert et al. 2015). For example, Caring for Country and Ranger Programmes promote multiple social benefits arising from participants’ ability to work on and access their Country (Altman and Whitehead 2003; Gorman and Vemuri 2012) and environmental enhancement through revegetation, weed management and regular, cool season burning (Garnett and Sithole 2007). Capturing the benefits of ICNRM within environmental reporting is important to ensure appropriate recognition and funding compensation for all Indigenous owners and managers.

Like other Indigenous and First Nations peoples around the world, Australia’s Indigenous peoples have important relationships with the environment that do not fully align with the prevailing conceptions of ecosystem services. To date, ecosystem services approaches have focussed on the flow of benefits from nature to people without recognition of the reciprocal responsibilities of people to care for the environment (Comberti et al. 2015). These responsibilities are embodied within concepts such as ‘Caring for Country’ in Australia, and stewardship concepts more generally (Stoeckl et al. 2021). Ecosystem services approaches in general and in the SEEA-EA also assume that service flows are separable (and that the separable flows are each measurable) which conflicts with the holistic worldviews of many Indigenous peoples (Sangha et al. 2018; Hill et al. 2021).

These issues are pertinent for the Yawuru people of the west Kimberley region of northern Australia. Operating through their development and investment company, Nyamba Buru Yawuru (NBY), the Yawuru people are one example of an Indigenous community who are active in the development of strategic approaches to support post-Native Title land management and governance (Potter et al. 2016). A specific emphasis of NBY’s work is on recognising the close linkages between *mabu liyan* and cultural and environmental management. Literally translated from Yawuru language as “good/strong spirit”, *mabu liyan* reflects a way of living well with all the things—Country, people, community, and culture—that together bring meaning and wellbeing to the lives of Yawuru people (Yu 2013). Such is the importance of the concept of *liyan* in NBY’s management, that a *liyan* framework has been developed (Yap and Yu 2016a) and implemented (Yap and Yu 2016b), to define the ambitions and framework for community programmes, cultural and language maintenance, land management, and economic development.

Healthy Country plays a central role in maintaining strong *liyan* for Yawuru people (Yu 2013; Yap and Yu 2016b) and as such NBY’s Environmental Services Unit (ESU) are engaging in the development of approaches to better understand the benefits provided by cultural and ecosystem services from across Yawuru Country, to both Yawuru people and to Yawuru natural capital. This has involved the assessment of how natures contributions to people (NCP) approaches align to NBY’s environmental management plans (e.g. Yawuru RNTBC 2013; Newman et al. 2019), and an investigation on how ecosystem accounting might practically support NBY to document bio-cultural values in a format relevant to management by NBY (Normyle et al. 2022b). For this assessment, a series of on-Country workshops were undertaken with the view to understand the potential usefulness of a SEEA-EA approach from the NBY perspective and representing the interests of the Yawuru people. This paper reports on the outcomes of these workshops and examines how they may inform the development of culturally relevant ecosystem accounts for stocks, flows and benefits on Indigenous-managed lands.

## Materials and methods

### Process of development

This work was supported by an ongoing collaborative partnership between NBY and The Australian National University (ANU) (NBY 2016, p. 8). NBY’s participation was exercised in all stages of this research including in the research conception, implementation, and subsequent analysis. Two NBY managers are co-authors of this paper and continue to provide guidance on the cultural-appropriateness and relevance of the research design and outputs.

Workshops were undertaken with NBY representatives in Broome in April 2018 and February 2020. NBY selected their participants to attend the workshops and as the operational company of the Yawuru Registered Native Title Holders Body Corporate, these members (hereafter, collectively termed ‘NBY managers’) were considered to hold the authority to speak on behalf of the Yawuru community, and Yawuru values (NBY 2020). While both workshops were conducted in English, where Yawuru-language terms such as *liyan* provided more appropriate context or meaning, these were used ahead of their English-translation.

The focus of the 2018 workshop was to bring together NBY managers and ecosystem accounting experts to explore the potential of ecosystem accounting to support NBY’s cultural and environmental management. Participants included senior managers of NBY (9, including co-authors DM and JM), and collaborators with expertise in the field of ecosystem accounting (5, including co-authors BD and MV) (Table 1).

**Table 1:** Participants included in the 2018 and 2020 ecosystem accounting workshop discussions

Participants	2018 workshop			2020 workshop		
	Male	Female	Total	Male	Female	Total
NBY managers	8	1	9	5	2	7
* Land & sea coordinators*	*4*	*1*	*5*	*2*	*1*	*3*
* Country managers*	*2*	*0*	*2*	*2*	*0*	*2*
* Other officers*	*2*	*0*	*2*	*1*	*1*	*2*
ANU researchers	2	0	2	1	1	2
Government representatives	2	1	3	0	0	0
**Total participants**	**12**	**2**	**14**	**6**	**3**	**9**

The 2-day 2018 workshop involved introductory presentations to set the context for the workshop, including introducing and explaining the SEEA-EA and examples of the framework’s application; ‘breakout’, small group discussions between participants; and whole-group discussions. During the workshop sessions, participants were asked to consider two overarching questions. First, whether they considered that the SEEA-EA could be used to measure the cultural and environmental benefits of NBY’s land management; and second, how ecosystem accounting could be applied practically to support the ESU in their management of Country. In evaluating these broad questions, a range of sub-questions were provided as a guide for participants (Table 2). Collaboratively, participants were asked to document their key ideas and responses onto a series of post-it notes that were transcribed by co-author GA as the primary method of data collection for the workshop.

**Table 2:** Summary of open-ended questions that guided the 2018 workshop discussions and justification of the use of each question

Question	Justification
How could an ecosystem accounting approach help NBY?	To gather broad perceptions of where ecosystem accounting might fit within NBY’s management priorities
Can the practical benefits of an ecosystem accounting system be defined?	Used to explore whether the perceived benefits discussed can be quantified as practical applications for SEEA Ecosystem Accounts
What are the cultural considerations for developing ecosystem accounts?	Used to prompt for participants’ perspectives on how cultural knowledge and values might, or might not, align to ecosystem accounting development
What is the best way to go about developing an ecosystem accounting system for NBY?	To initiate a discussion about capacity, resources, datasets and the potential inclusions for accounts developed on Yawuru Country
What existing data and approaches can NBY draw upon?	Used to link ecosystem accounting to NBY’s existing management frameworks, such as geospatial modelling work and on-Country monitoring
How would NBY benefit from leading a ‘proof-of-concept’ in this area?	Used to directly link discussion to the broader context of Indigenous inclusion within environmental accounting development in Australia

Based on NBY’s interest in ecosystem accounting during the 2018 workshop, a follow-up workshop was undertaken in February 2020. This workshop was attended by senior NBY managers (7, including co-authors DM and JM). Co-authors BD and AN were present during these workshop sessions. Of the total 9 participants present at the 2020 workshop, 5 participants (4 from NBY and 1 from ANU) were present during the 2018 workshop.

The 2020 workshop reviewed the key themes raised during the 2018 workshop and provided a basis for further discussions with NBY’s managers regarding their attitudes towards ecosystem accounting.

The sessions followed a semi-structured approach, with participants asked two questions:

1. How do you think ecosystem accounts could support management?
2. How do you envisage a cultural dimension being included within an experimental accounting approach?

Discussion was allowed to flow naturally based on the responses provided, with this semi-structured approach ensuring that conversational rapport was retained with knowledge holders, while allowing new information to arise spontaneously (Huntington 2000). With permission from the workshop participants, the 2020 workshop was recorded and subsequently fully transcribed by co-author AN.

### Data distillation and organisation

We distilled the data obtained from both workshops using thematic analysis methods (Althor et al. 2018). The data corpus consisted of (transcribed) data gathered on notes from participants during the 2018 workshop, and transcribed data recordings of the 2020 workshop discussions. From these data, we used inductive analysis to create a codebook of participant responses (Braun and Clarke 2006). Separate code books were created for the 2018 and 2020 workshop data, although the similarity of the themes discussed resulted in both datasets being nested under the same overriding themes. Coding was undertaken using NVIVO 12, as per Richards (2005).

Coding required three data screening phases following Frith and Gleeson (2004). Between each screening phase, all data were completely un-coded.

1. An initial screening of data for the purposes of identifying themes and sub-themes, and organisation of themes into a draft code book.
2. A second screening of data to test the precision of, and to refine, the code book.
3. A final screening of data, where data were re-coded using the final, refined code book.

Deductive, semantic thematic analysis methods were used to analyse, create thematic patterns and infer meaning from the coded datasets (Braun and Clarke 2006). We coded data by interpreting its relevance to the themes used in the workshops: opportunities; uses; challenges; and requirements. Data extracts were not necessarily limited to a single theme or sub-theme. For example, we determined that several data extracts captured in the workshop under *requirements* were most relevant to the *challenges* theme, while others were relevant to both. Themes were then further broken up into sub-themes, depending on prevalence. All data interpretations were assessed by NBY’s managers (co-authors DM and JM) to ensure an accurate representation of the workshop discussions from NBY’s perspective.

## Results

**Fig. 2:**
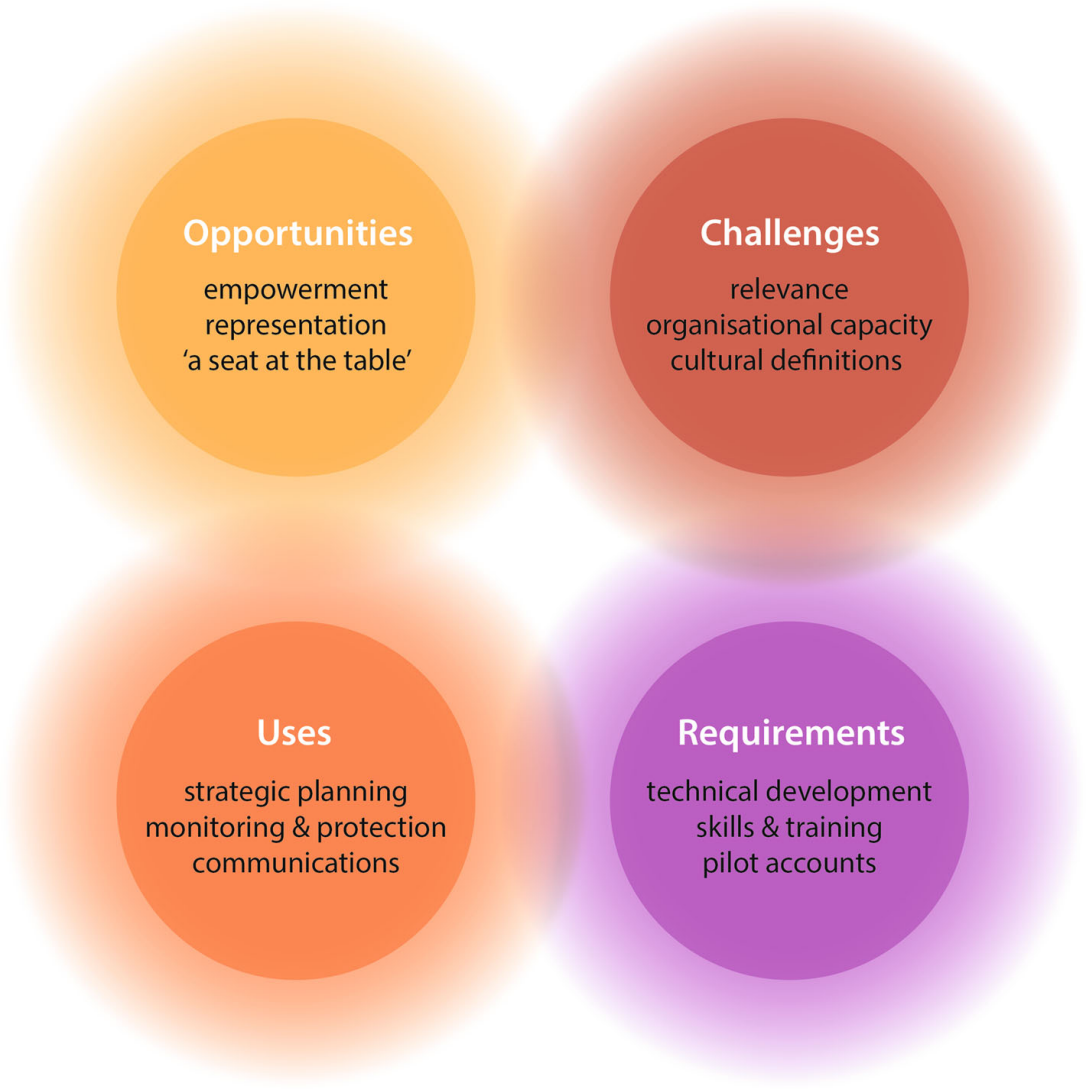
Key themes from data gathered during ecosystem accounting workshops. Four main themes were identified during the workshops, with each consisting of three sub-themes. The blurred edges of the thematic circles represent the inter-relatedness of the themes identified, as the anticipated challenges to an ecosystem accounting approach influenced both the account requirements and the opportunities for ecosystem accounting, which, in turn influenced the uses discussed

In answer to the question of whether ecosystem accounting could support NBY’s cultural and environmental management, four themes—opportunities, challenges, uses, and requirements—were identified from the workshop discussions (Fig. 2). These themes were divided into several sub-themes, which are discussed in the following sections.

### Opportunities

The most commonly discussed opportunities revolved around using ecosystem accounting as a tool to empower Indigenous peoples, both for Yawuru people specifically and for Indigenous peoples elsewhere in Australia and around the world. By playing a leading role in the development of a culturally inclusive application of ecosystem accounting, Yawuru people, it was anticipated, would have an opportunity to ensure that (1) non-Western values are represented within the SEEA framework; and (2) SEEA-based accounts can be used to support the aims of Traditional Owners as part of Australia’s broader National Environmental Economic Accounting Strategy and Action Plan (DoEE 2018). It was discussed that in developing a unique ecosystem accounting system for Yawuru Country, NBY could use traditional knowledge to take the first steps towards modelling culturally inclusive ecosystem accounting frameworks, extending the current SEEA-EA. Such frameworks could be transferred to and adapted by other Indigenous peoples both in Australia and globally and included in updates to the SEEA-EA.

Workshop participants similarly suggested that the development of an Indigenous ecosystem accounting approach could potentially increase the power and influence of Indigenous peoples in decision-making processes at a local and region scale; particularly, in negotiations where a range of stakeholders are involved. One NBY manager commented:

Knowing that the value of the accounts is as a commodity that gives a seat at the table... that comes back to the fact that Indigenous people aren’t intangible, and the Indigenous worldview can’t be dismissed as an intangible asset.

Participants commented that an inclusive ecosystem accounting approach could be a key opportunity to embed Indigenous values within broader planning and policy regimes and highlight how Yawuru Country has changed following a return to Indigenous management.

### Uses

The discussed uses for ecosystem accounting centred on the system’s potential as a tool to enhance strategic planning for NBY's ESU. Participants observed that an accounting system could be used to consolidate information about ecosystem services, and particularly cultural services, while also integrating the connectivity of Yawuru social and cultural wellbeing with Country. This information was viewed as potentially valuable for the ongoing monitoring, evaluation, and communication about current practices—in particular the management of cattle and their threats to culturally sensitive *jila* (springs). One NBY manager commented:

The accounts could help to show different impacts from the cattle... it could show us how impacted sensitive habitat types are and that might help to work out focus areas for management.

Participants agreed that developing an ecosystem accounting system would allow for the consistent measurement of the upstream and downstream effects of land-use practices and change over time against a baseline that the accounts could help determine. This could be used to maximise the sustainability of land use, and enhance NBY’s ability to protect threatened, commercially important, and emblematic species.

### Challenges

The challenge of assuring social and cultural relevance for account development was a key concern for NBY. The organisation’s managers noted that maintaining local relevance to ensure community buy-in would be crucial, as would ensuring that any accounts developed are inclusive of, and provide tangible benefits to, the broader Yawuru community. One NBY manager commented:

For this process to be meaningful to the community then we need to think about why we are developing this... developing the accounts needs to be about the community.

In this, challenges were identified around precedent. Currently there is no explicit approach for introducing Indigenous cultural values and knowledge in the SEEA-EA. As such it would be necessary for NBY (supported by partners such as the ANU and the Kimberley Land Council) to develop ecosystem accounting approaches from the ground up, which would be a significant burden (although this was also viewed as a potential opportunity for the Yawuru people to lead in this space). There would also need to be approaches setup to support the inclusion of cultural values within a set of experimental accounts, as consideration of cultural values is currently lacking in SEEA-EA applications.

NBY’s managers also emphasised the importance of *liyan* (Yu 2013) when defining cultural values in the Yawuru context. *Liyan* was linked to rights to access and conduct activities such as hunting, fishing and ceremony on Country, in addition to the environmental health of the landscape and social relationships within the community. This was captured by one NBY manager:

Culture, that *liyan* feeling is about rights and access to Country and the rights of Yawuru people to hunt and take and enjoy Country.

When considering how *liyan* may be embedded within ecosystem accounts, concerns were raised about the lack of any pre-existing metrics to quantify benefits from ecosystems that would be of equivalence to *liyan.* Wellbeing, a good proxy indicator (e.g. Sangha et al. 2015), is mentioned in the opening sentence of the Introduction to the SEEA-EA (UN 2021, p. 2), but it is not defined nor included in the conceptual model of the SEEA-EA (UN 2021, p. 28). Wellbeing is also mentioned in the description of the ecosystem services related to non-use values and to “ecosystem and species appreciation” (UN 2021, p. 134) and in the section on indicators, but no wellbeing metric is proposed.

### Requirements

It was identified that a key requirement for the NBY-ANU partnership would be to engage in developing a series of pilot ecosystem accounts to test the usefulness of biophysical and cultural accounting applications. This could help to demonstrate the usefulness of accounts practically to other NBY members and the community.

Requirements were also raised around organising project management for ecosystem accounting, particularly in relation to the tension between ensuring that Indigenous governance is given primacy (e.g. Yu 2012), while also recognising that NBY have limited resources and many competing responsibilities. It was established that to be beneficial, an ecosystem accounting system would need to deliver tangible, measurable, and policy appropriate information, supporting Indigenous rights, responsibilities, and self-governance.

## Discussion

Indigenous peoples’ knowledge and values are underrepresented in environmental and economic assessment, and it is crucial that any quantification of ecosystem services on Indigenous lands be Indigenous-led and customised to ensure Indigenous perspectives are not marginalised from reporting (Sangha et al. 2018). Through discussions with NBY, an Indigenous organisation tasked with administering the rights of the Yawuru Traditional Owners, the opportunities and challenges to using a SEEA-EA approach to support the organisation’s strategic management were canvassed. This work is important in placing the needs of an Indigenous organisation at the forefront of developing ecosystem accounting approaches and should contribute to the broader recognition of Indigenous knowledge and values within the SEEA-EA, where they are currently lacking.

### Towards a SEEA-EA approach that supports management of Country

**Fig. 3:**
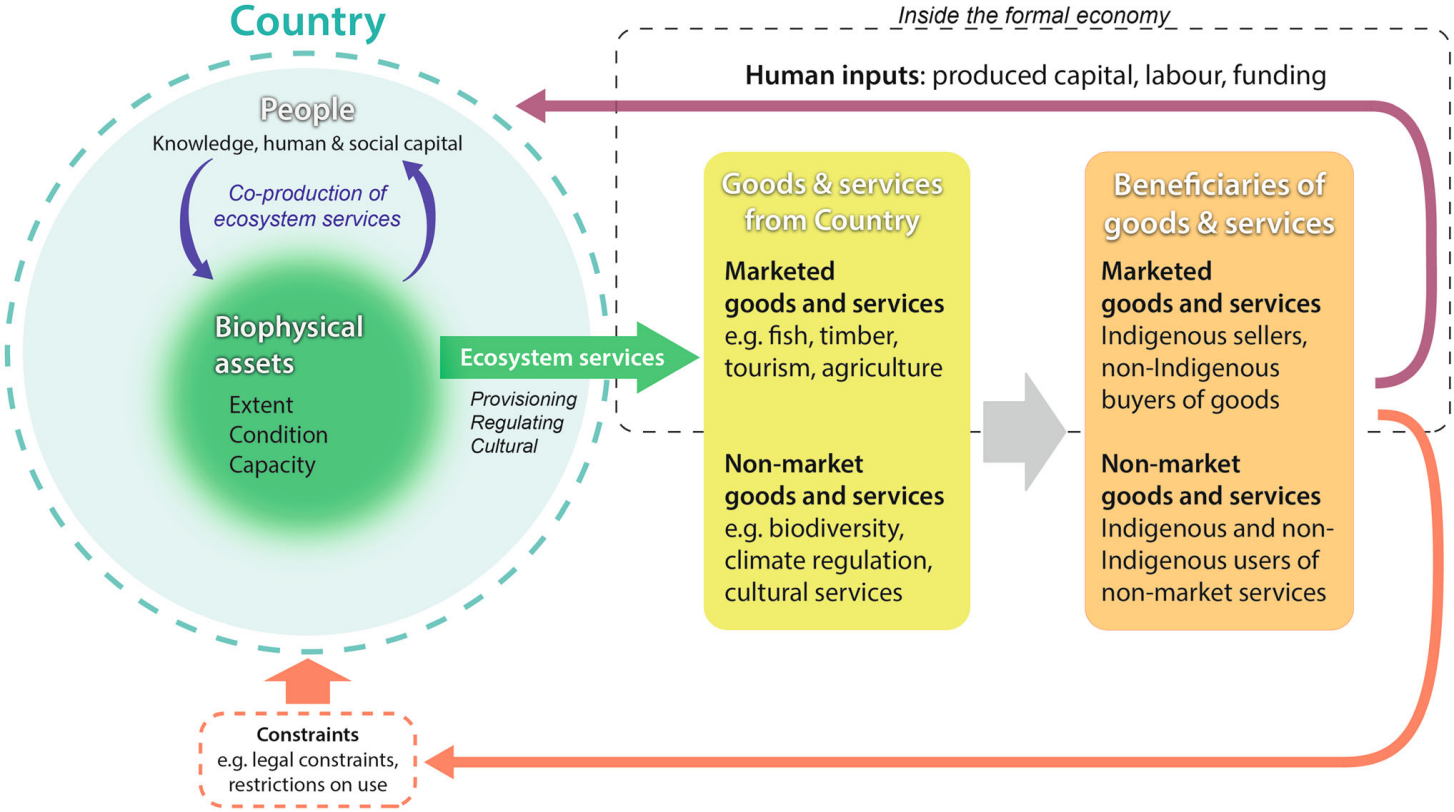
Conceptual model of service flows from and to Country adapted from the SEEA-EA. Country comprises of both human and biophysical assets, where a two-way reciprocal relationship co-produces ecosystem services. Our figure differs from the visualisation in the SEEA-EA (UN 2021, p. 28) with flows from the beneficiaries, both Indigenous and non-Indigenous people, returning to the biophysical assets. Biophysical assets produce ecosystem services that can be traded within formal markets (cattle, fish, timber), as well as those that are not (biodiversity values, aesthetic services, cultural and spiritual values)

Our case study identified that there may be practical opportunities for an ecosystem accounting approach to support NBY’s strategic management of Yawuru Country. By enabling the current state of a particular area consisting of various ecosystems to be assessed spatially in a regular and consistent manner, ecosystem accounts can support an evaluation of the current and potential costs and benefits of varied management approaches (Warnell et al. 2020). If, in the Yawuru and other Indigenous contexts this area, known as the *ecosystem accounting area* in the SEEA-EA (UN 2021, p. 48), encompasses the area of land recognised as Indigenous owned or managed, accounts could provide valuable information about how that area has changed under Indigenous management. Figure 3 shows a conceptual model for how the SEEA-EA may be adapted to reflect an Indigenous-managed landscape in the Yawuru context.

In Fig. 3, Indigenous land, or the *ecosystem accounting area*, is visualised as Country, the living place where, for Indigenous people, intimate physical, ecological, emotional, spiritual, social, and cultural connections exist (Kwaymullina 2005). The biophysical characteristics of Country are described by extent, condition and capacity. People are also included as a core part of Country, as Indigenous knowledge is a key to maintaining “healthy Country”, and “healthy Country” is inextricably linked to Indigenous peoples’ wellbeing (Stoeckl et al. 2021). Determining measures for extent, condition, and capacity should consider Indigenous peoples’ knowledge about the biophysical assets that encompass Country, and the role of Indigenous peoples in the co-production of ecosystem services.

The SEEA-based model in Fig. 3 also shows that the flows of goods and services from Country extend to both Indigenous and non-Indigenous peoples as ecosystem service flows. These goods and services may be sold in markets (where formal markets exist) or used without a monetary transaction (outside of markets and not captured in SNA reporting). For example, cattle are sold in market, while hunting by Indigenous people is an example of a service of largely non-market benefits (recreational and symbolic benefits), alongside benefits that can be valued monetarily using exchange values (e.g. as a food provisioning service). Consistent with other case studies in ecosystem accounting, the biophysical land assets that comprise Country, and the services produced from Country, may be valued in monetary terms as “exchange values” (Remme et al. 2015). In this instance, the monetary value of the ecosystem asset(s) as an exchange value could be calculated based on the present value of the flow of ecosystem services estimated in monetary terms (as an exchange value) (UN 2021, p. 177). It is recognised, however, that such values would not account for the substantial intangible and relational values of Country. The challenges for valuing the monetary contribution of some cultural services (such as symbolic species and a sense of place), given the intangible and incommensurable nature of these values (Stoeckl et al. 2018), are recognised as a key area where further development is needed to align Indigenous views on the benefits arising from Country with the SEEA-EA conceptually. Valuation is an area given high priority in the SEEA-EA research agenda.

### Linking cultural values with the SEEA-EA

Considering how principles of relationality, reciprocity, and wellbeing, which are central to the livelihoods and perspectives of many Indigenous communities (IPBES 2019), might align within the existing measurement and classification systems of the SEEA-EA is crucial, as these principles underpin the value flows between people and the environment for many Indigenous communities. In the following sections, we propose two accounting approaches to better integrate these principles within ecosystem accounts in the context of the SEEA-EA.

#### Assessing bio-cultural ecosystem condition

Ecosystem condition accounts are part of the SEEA-EA, containing aggregated statistical information about the overall abiotic and biotic quality of an ecosystem (Maes et al. 2020). Conventionally, condition accounts use biophysical indicators which are assessed relative to a pre-industrialisation ‘baseline’ (UN 2021, p. 115). This approach is not appropriate in an Indigenous context, where human occupation (and hence interactions) with ecosystems has been documented for thousands of years prior to European industrialisation (Clarkson et al. 2017). One way to address this challenge may be the development of indicators for the relative bio-cultural condition of ecosystem assets that would complement existing ecological condition metrics. The ‘bio-cultural condition indicators’ would derive a cultural assessment of the landscape health as determined by Traditional Owners, or those deemed to hold the appropriate cultural authority to make the assessment. This would ensure that the overall condition of assets explicitly integrates Indigenous knowledge about landscape health into biophysical reporting and provides a non-western perspective about the health of Country.

Table 3 provides an indicative example of a bio-cultural condition account for the Yawuru case study. The opening and closing stock condition are reported in qualitative terms aligned to the good to bad ‘traffic light’ system NBY uses for reporting. The condition account allows scope to link net change to human-induced (anthropogenic) or environmental change (managed and unmanaged expansion and reduction in the SEEA-EA) in qualitative terms. Since for Yawuru people, the health of Country is aligned closely to notions of *liyan*, the bio-cultural condition score could be derived as the relative *liyan* that the Yawuru Traditional Owners associate with each ecosystem asset. While qualitative, the consistency that underpins ecosystem accounting (e.g. Wentworth Group of Concerned Scientists 2016) would be embedded within the bio-cultural condition assessment by drawing on historical knowledge of the health of Country (i.e. passed on through Traditional Owners), and the recording of information using simple consistent indicators such as a Likert scale (Albaum 1997).

**Table 3:**
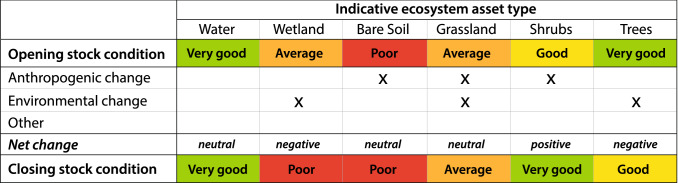
Example bio-cultural condition account. The account records the relative bio-cultural condition at opening and closing for each ecosystem asset type as inferred by knowledge holders. Positive and negative change can be qualitatively attributed to anthropogenic and/or environmental changes such as weeding, habitat protection or clearing for development (anthropogenic) and rainfall, wildfire, or drought (environmental). Note that the values indicated here are for demonstration purposes only

It is anticipated that a similar method for developing a bio-cultural condition account would be possible for other groups of Traditional Owners and Indigenous land managers, at least in the Australian context. Indigenous Protected Area (IPA) reporting in Australia already requires Indigenous managers to develop indicators for protected area assessment that encompass a “mix of environmental, cultural (including strengthening and integrating Indigenous knowledge with Western Science), social (community capacity and wellbeing) and economic (jobs and Indigenous enterprises) factors” (NIAA 2015). The guidelines recommend using methods that involve the ‘right’ people who can ‘speak for Country’ and employing a traffic light reporting system as visualised in the example Table 3.

While such data could be used to inform a relatively consistent approach to compiling bio-cultural condition accounts in other case studies, any aggregation of such data (for example at a national level) would be challenging for three reasons. First, because assessment of bio-cultural condition is dependent on a specific community/Traditional Owner groups’ interpretation of their land and sea, it would be inappropriate and a misuse of knowledge to extend inferences to other areas not managed by that group. Second, since the use of compatible reference levels (e.g. through a common reference condition) is needed to underpin the aggregation process of ecosystem condition indicators in the SEEA-EA (UN 2021, p. 315), it may not be possible to invoke a ‘common reference condition’ across different groups of Indigenous Owners and managers. Thirdly, it is unclear how a national-scale account would support national-level decision making. Consequently, we recommend that bio-cultural condition accounts be developed foremost as a tool to support Indigenous Owners and managers to report on cultural and biophysical change as it occurs across their Country to inform local management priorities.

#### Accounting for services to ecosystems

Considering how (and if) the ICNRM services provided by Indigenous management practices could be classified within the SEEA-EA is important. Currently, the SEEA-EA only discusses those ecosystem services delivered by ecosystems to society (e.g. UN 2021, p. 28) and the traditional ecosystem services framework does not account for the regulating, protecting, and enhancing services that humans may provide to ecosystems (Comberti et al. 2015). Indigenous relationships with the environment commonly emphasise a two-way reciprocal relationship whereby people look after the land, and the land correspondingly looks after people (Stoeckl et al. 2021). Although the SEEA Central Framework includes environmental protection and resource management actions and expenditures which are aligned with the notions of people providing services to ecosystems (UN 2014a, p. 116), this has never been applied in an Indigenous context.

The allowance of auxiliary accounts under the SEEA–EA offers a useful starting point to incorporate information on the services Indigenous land holders provide to ecosystems. Table 4 provides an indicative example of a supply table populated according to services to ecosystems. While a corresponding use table could be developed, this would require assumptions regarding the extent to which the services described are *used* by ecosystems. Since developing metrics for this is likely to be challenging (Rillig et al. 2021), considering the extent to which it is appropriate to infer use based on supply, or to provide supply data independent of use (noting that the SEEA-EA defines ecosystem services based on their use), is an area in need of further development.

**Table 4:** Example services to ecosystems supply table. The account records the time spent providing protecting, enhancing, and restoring services to ecosystems in hours. Note that the values indicated here are for demonstration purposes only

Supply table	Indicative ecosystem asset type						Total supply (hours)
Service to ecosystems	Water	Wetland	Bare	Grassland	Shrubs	Trees	
Protecting services	*	15	–	10	–	30	55*
* Cultural prohibitions on species*	*						*
* Weeding and culling*				*10*		*5*	***15***
* Habitat protection (e.g. fencing)*		*15*				*25*	***40***
Enhancing services	–	–	–	–	–	–	
* Cultivation*							
* Translocation*							
Restoring services	50	*	–	50	70	5	175*
* Nutrient release (burning)*					*30*		***30***
* Habitat (re)construction*							
* Eco-cultural harvesting*	*50*	*		*50*	*40*	*5*	145*
**Total service supply**	50*	15*	**–**	**60**	**70**	**35**	230*

*Categories such as *cultural prohibitions on species* and *eco-cultural harvesting* may not be readily quantified with a temporal reporting metric, with supplementary qualitative clarifications more appropriate

In Table 4, supply is recorded by ecosystem type and services include those human activities that enhance, protect and restore ecosystems following Comberti et al. (2015). Although the units of measurement of services to ecosystems could include exchange values (e.g. wages in dollars), used independently monetary metrics are unlikely to account for the importance of activities that are undertaken outside of formal markets (Sangha et al. 2017). Therefore, the example in Table 4 quantifies services to ecosystems as the time spent conducting the services (e.g. the time spent working on Country), and also allows scope of a qualitative clarification where the temporal classification may not be appropriate to quantify the service identified (e.g. cultural prohibitions on species may only apply to some areas or times of year, yet provide an ongoing protecting service).

### Next steps

The engagement of NBY with ecosystem accounting development has great potential to practically demonstrate Indigenous leadership in the advancement of inclusive environmental governance frameworks. As a tool promoted by Australia’s governments (e.g. DoEE 2018), the application of ecosystem accounts by Indigenous organisations will be an important step to signify improved representation of cultural knowledge within environmental and economic reporting. However, further work is required to develop the SEEA-EA so that accounts can be produced that are useful to Indigenous peoples in Australia and around the world. First, Indigenous values should be included as a new item on the SEEA-EA research agenda. This will ensure that focus is given to addressing challenges, such as the measurement and classification of cultural assets and services, and the scaling of these data from local to global contexts. While much can be learned from existing ecosystem services and NCP approaches (e.g. Sangha et al. 2017; IPBES 2019), the specific emphasis of ecosystem accounting on the consistent valuation of stocks, flows and benefits across spatial and temporal scales requires conceptual development to consider how Indigenous perspectives on wellbeing and reciprocal benefit flows may be measured and applied across different case studies.

Indigenous peoples also need to be included as part of the United Nations (UN) processes governing the SEEA-EA via their inclusion in the UN Committee of Experts on Environmental-Economic Accounting (UNCEEA) (Normyle et al. 2022a). Such representation is crucial to guiding future development, informing collaborations and networks and fostering development of mutual benefit. Globally, Indigenous peoples have a leading role to play in shaping alternative paradigms to mainstream economic models that better encompass a relational understanding of human sustainability with the environment (Yu 2012). Since considering context-specific appropriateness has been a common failure in the implementation of Indigenous policy in Australia and globally (Westbury and Dillon 2019), prioritising further engagement with Indigenous peoples and organisations will be key to developing standards, datasets, and methods to identify commonalities and differences across regional and global contexts.

## Conclusions

To date, there has been limited inclusion of Indigenous peoples and perspectives in the development of the SEEA. Considering Indigenous perspectives is crucial for ensuring Indigenous peoples, knowledge and values are considered in government and local decision making. If the SEEA is to be useful for, and used by, Indigenous peoples then the framework needs to reflect the values and needs of Indigenous peoples and organisations. The case study with NBY has shown that the SEEA-EA has the potential to assist the ambitions of an Indigenous organisation, at least in this case, and probably for others too.

Practically, implementation of ecosystem accounting for NBY could improve monitoring capability and enhance the communication of cultural, environmental, and economic information in a format relevant to both NBY and government. Theoretically, further work is needed to ensure that the SEEA-EA accounts for Indigenous knowledge and management. In particular, considering how two-way value flows may be represented and applied in SEEA-EA case studies, and how these flows can be related to wellbeing requires attention. Further engagement with Indigenous peoples and organisations through a range of applications globally and Indigenous peoples' representation as part of the SEEA’s governance is crucial for bridging a range of perspectives towards the classification and measurement of assets, benefits and flows in accounting.

## Footnotes

1 In this paper, the term *Country* refers to the living place where, for Indigenous peoples, intimate physical, ecological, emotional, spiritual, social, and cultural connections exist (Kwaymullina 2005). *Country* refers to all the elements of spaces and places and their interactions, both tangible and intangible.
